# Rap1a Overlaps the AGE/RAGE Signaling Cascade to Alter Expression of α-SMA, p-NF-κB, and p-PKC-ζ in Cardiac Fibroblasts Isolated from Type 2 Diabetic Mice

**DOI:** 10.3390/cells10030557

**Published:** 2021-03-04

**Authors:** Stephanie D. Burr, James A. Stewart

**Affiliations:** Department of BioMolecular Sciences, School of Pharmacy, University of Mississippi, Oxford, MS 38677, USA; jastewa7@olemiss.edu

**Keywords:** diabetes, AGE/RAGE, Rap1a, inflammation, oxidative stress, heart, fibroblasts, myofibroblasts

## Abstract

Cardiovascular disease, specifically heart failure, is a common complication for individuals with type 2 diabetes mellitus. Heart failure can arise with stiffening of the left ventricle, which can be caused by “active” cardiac fibroblasts (i.e., myofibroblasts) remodeling the extracellular matrix (ECM). Differentiation of fibroblasts to myofibroblasts has been demonstrated to be an outcome of AGE/RAGE signaling. Hyperglycemia causes advanced glycated end products (AGEs) to accumulate within the body, and this process is greatly accelerated under chronic diabetic conditions. AGEs can bind and activate their receptor (RAGE) to trigger multiple downstream outcomes, such as altering ECM remodeling, inflammation, and oxidative stress. Previously, our lab has identified a small GTPase, Rap1a, that possibly overlaps the AGE/RAGE signaling cascade to affect the downstream outcomes. Rap1a acts as a molecular switch connecting extracellular signals to intracellular responses. Therefore, we hypothesized that Rap1a crosses the AGE/RAGE cascade to alter the expression of AGE/RAGE associated signaling proteins in cardiac fibroblasts in type 2 diabetic mice. To delineate this cascade, we used genetically different cardiac fibroblasts from non-diabetic, diabetic, non-diabetic RAGE knockout, diabetic RAGE knockout, and Rap1a knockout mice and treated them with pharmacological modifiers (exogenous AGEs, EPAC, Rap1a siRNA, and pseudosubstrate PKC-ζ). We examined changes in expression of proteins implicated as markers for myofibroblasts (α-SMA) and inflammation/oxidative stress (NF-κB and SOD-1). In addition, oxidative stress was also assessed by measuring hydrogen peroxide concentration. Our results indicated that Rap1a connects to the AGE/RAGE cascade to promote and maintain α-SMA expression in cardiac fibroblasts. Moreover, Rap1a, in conjunction with activation of the AGE/RAGE cascade, increased NF-κB expression as well as hydrogen peroxide concentration, indicating a possible oxidative stress response. Additionally, knocking down Rap1a expression resulted in an increase in SOD-1 expression suggesting that Rap1a can affect oxidative stress markers independently of the AGE/RAGE signaling cascade. These results demonstrated that Rap1a contributes to the myofibroblast population within the heart via AGE/RAGE signaling as well as promotes possible oxidative stress. This study offers a new potential therapeutic target that could possibly reduce the risk for developing diabetic cardiovascular complications attributed to AGE/RAGE signaling.

## 1. Introduction

Cardiovascular disease, such as heart failure, is a common complication for patients suffering from type 2 diabetes mellitus (T2DM). Heart failure can be attributed to left ventricle (LV) hypertrophy, which results from increased oxidative stress, inflammation, and cell-mediated remodeling of the extracellular matrix (ECM) [1,2]. A key cellular component within the cardiac tissue contributing to these effects are cardiac fibroblasts, which under healthy conditions, are primarily responsible for maintaining the ECM, and under pathological conditions can trigger cardiac fibroblast transition into myofibroblasts, as indicated by increased alpha smooth muscle actin (α-SMA) expression [3,4,5]. In addition, cardiac fibroblasts can contribute to inflammation and increased ROS (reactive oxygen species) production within the cardiac tissue [6,7,8,9]. The combination of increased ECM production, inflammation, and elevated ROS has been shown to exacerbate cardiac fibrosis, a key causative factor in heart failure [4,6,7,9,10]. Furthermore, the generation and regulation of both these mechanisms have been linked to the AGE/RAGE signaling cascade.

Advanced glycated end products (AGEs) exert influence on cellular signaling by binding and activating the receptor for advanced glycated end products (RAGEs). Overtime, AGEs, formed through non-enzymatic reactions between a sugar and protein, accumulate within the body, and this process is accelerated under diabetic conditions [11]. Within the ECM, AGEs can form crosslinks between matrix proteins, impacting tissue rigidity as well as stimulating intracellular signaling [12,13]. The signaling cascades elicited by AGEs produce changes in expression of proteins associated with a wide range of effects varying from oxidative stress/inflammation to ECM remodeling [13,14,15,16]. For example, AGEs have been shown to increase the activity and expression of protein kinase C zeta (PKC-ζ) and NADPH oxidase (NOX), which are major components of the nicotinamide adenine dinucleotide phosphate (NADPH) oxidase complex [17,18,19]. The increase in NADPH oxidase activity, triggered by AGE/RAGE, results in increased ROS production, which can lead to increased expression of nuclear factor kappa-light-chain-enhancer of activated B cells (NF-κB), a transcription factor linked to inflammation [20,21]. NF-KB exhibits a dynamic role within the RAGE signaling cascade, whereby phosphorylation of NF-KB can be triggered directly by AGE/RAGE signaling or indirectly by ROS production as a product of increased AGE/RAGE signaling, extracellular signal related-kinase 1/2 (ERK1/2) activity, and/or tumor necrosis factor alpha (TNF-α) expression [15,21,22,23,24]. Increases in AGE/RAGE mediated ROS and signaling proteins (NF-κB, PKC-ζ, and ERK1/2) have been shown to alter the expression of superoxide dismutases (SODs), where SOD expression has been linked to both PKC-ζ and ERK1/2 activity in neural cells [15,20,25,26,27]. Furthermore, many of these signaling and ROS proteins have been linked to impact expression of proteins associated with ECM remodeling. For example, Lin et al., 2006 demonstrated exposure to AGEs induced ROS production that further increased fibronectin synthesis, via ERK activation, within rat mesangial cells and treatment with SODs appeared to reduce the synthesis of fibronectin and ERK activaiton [14]. These studies highlight the complexity of the AGE/RAGE signaling cascade and the numerous signaling molecules involved. To add further complexity, we have identified a small GTPase, Rap1a, which we suspect overlaps the AGE/RAGE signaling cascade to alter the expression of RAGE associated signaling proteins to impact the downstream effect of the signaling cascade [28].

Repressor/activator protein 1a (Rap1a) is a small GTPase of the Ras superfamily and is associated with multiple organ systems and signaling pathways [29]. Specifically, within the cardiovascular system, Rap1a is involved in the development and function of the heart, where it acts as a molecular switch linking extracellular signals to intracellular responses [29,30]. Rap1a activation can be induced by the exchange protein activated by cyclic AMP (EPAC) to elicit signaling cascades impacting effector proteins involved with both the cardiovascular system and regulating oxidative stress [31,32]. In the cardiovascular system, Rap1a has been documented to impact fibroblast migration and proliferation via activation of ERK1/2 and PKC [28,33]. In addition, a study conducted by He et al., 2010 showed that Rap1a in conjunction with ERK1/2 promoted hypertrophy in neonatal ventricular myocytes [34]. Rap1a has also been verified to impact oxidative stress by interacting and binding to p22^phox^, a subunit of the NADPH oxidase complex, which has been linked to hypertrophy in vascular smooth muscle cells [35,36]. Additionally, Xia et al., 2007 demonstrated that p22^phox^ expression was increased by PKC-ζ activity under hyperglycemic conditions [37]. Overall, these studies highlighted the link between Rap1a and effector proteins, such as ERK1/2 and PKC, and their impact on oxidative stress and ECM remodeling in the cardiovascular system. Furthermore, these signaling proteins have also been demonstrated to be activated by AGE/RAGE signaling [33,38,39].

There is very little information regarding the role of Rap1a in diabetes-induced AGE/RAGE signaling. This study aims to better understand the effects of Rap1a on signaling proteins associated with the AGE/RAGE cascade. Therefore, we hypothesized that Rap1a crosses the AGE/RAGE cascade to alter expression of AGE/RAGE associated signaling proteins in cardiac fibroblasts in type 2 diabetic mice. To accomplish this, we utilized isolated cardiac fibroblasts from genetically different mice and exposed the cells to different pharmacological modifiers to manipulate AGE/RAGE signal cascade effector proteins as well as Rap1a expression, and thus, affording us the ability to assess the impact of Rap1a on the AGE/RAGE signaling cascade. These results determined that Rap1a interacted with the AGE/RAGE cascade to modify signaling proteins that could affect downstream mechanisms regulating oxidative stress and ECM remodeling within cardiac fibroblasts.

## 2. Materials and Methods

### 2.1. Animal Models

Male *Lepr^db^* (db/db model) type 2 diabetes mellitus mice (BKS Cg-*DOCK7^m^* +/+ *Lepr^db^*/J, Jackson Labs; JAX# 00642) were utilized in this study. A point mutation in the leptin receptor within the db/db mouse led to a nonfunctional leptin receptor. This mutation resulted in obesity and insulin resistance to cause the development of hyperglycemia by 8 weeks of age and by 12 weeks of age overt diabetes. Heterozygous male mice (non-diabetic) were used as lean controls. In addition, homozygous RAGE knockout (RKO) mice were used for this study. Generation of RKO mice was achieved by flanking exons 2–7 with two *loxP* sites in the same orientation and exposure to Cre recombinase, via breeding with Cre deleter mice, resulted in the deletion of the *loxP* sites and exons 2–7 [40,41,42,43]. This deletion results in a constitutive, global loss of RAGE mRNA expression and, in turn, knocking out RAGE signaling within these mice. Furthermore, a reversely orientated transcriptional EGFP reporter gene was inserted into intron 7 for confirmation of RAGE exons 2–7 deletion. EGFP PCR genotyping reactions are performed as a positive control for RAGE knockout mice. Documentation of loss of genomic RAGE and expression of EGFP was presented in Burr et al. 2020 (https://doi.org/10.6084/m9.figshare.11299253, accessed on 23 March 2020) [5]. RKO mice were crossbreed with heterozygous (non-diabetic) mice to generate RAGE knockout non-diabetic (non-diabetic RKO) and diabetic (diabetic RKO) mice [5,40,41]. Breeder RAGE knockout mice were a generous gift from Dr. Pamela Lucchesi and Dr. Angelika Bierhaus. Male Rap1a knockout mice (Rap1a KO) were utilized within this study as well. The Rap1a mouse model was generated by inserting a neomycin resistant gene downstream of exon 4 of RAP1A in the opposite (3′–5′) orientation. In order to insert the resistance gene, a targeting vector (a 0.95 kb *Pyull-Ndel* fragment) was used to disrupt Rap1a mRNA expression [44]. Breeder Rap1a mice were a generous gift from Dr. Maqsood Chotani and Dr. Lawrence Quilliam.

### 2.2. Animal Care

Animals were housed under standard environmental conditions with 12 h/12 h light/dark cycle and maintained on commercial mouse chow and tap water ad libitum. All studies conducted followed the principles of the National Institutes of Health “Guide for the Care and Use of Laboratory Animals,” (NIH publication No. 85–12, revised 1996). The animal protocol was approved by the University of Mississippi Institutional Animal Care and Use Committee (protocol #17-024). Anesthesia for euthanasia at the experimental endpoint of 16 weeks of age was caused by CO_2_ inhalation followed by cervical dislocation, which served as a secondary mechanism for euthanasia. Body weight and non-fasting blood glucose levels were determined (Table 1), followed by opening the chest cavity and quickly removing the heart for further cellular biochemical experiments.

### 2.3. Cardiac Fibroblast Isolation

Hearts were removed from mouse chest cavity, atria and great vessels were dissected away, and the ventricles were weighed (Table 1). Under sterile conditions, hearts were cut into approximately 5-mm sections and placed in a collagenase–trypsin enzymatic solution (0.1% Trypsin, Gibco; 100 U/mL collagenase II, Worthington Biochemical, Lakewood, NJ, USA) [4]. Fibroblasts were continually mixed until hearts were broken down into a single cell suspension. The cell suspension was centrifuged and resuspended in high glucose Dulbecco’s modified Eagles medium (DMEM) media (high glucose media) containing 4.5 g/L glucose, L-glutamine, sodium pyruvate, and supplemented with 14.9 mM HEPES, 14.2 mM NaHCO3, 1% L-glutamine, 0.02% Primocin™ (Thermo Fisher, Waltham, MA, USA), and 15% heat-inactivated fetal bovine serum (FBS) for 24 h in an incubator (5% CO_2_; 37 °C). After 24 h, fibroblasts were washed with appropriate media (non-diabetic and Rap1a fibroblasts: low glucose; 1 g glucose/L and diabetic fibroblasts: high glucose; 4.5 g glucose/L) three times and then incubated at 37 °C. Two to three hearts were used per one cardiac fibroblast isolation, where one isolation equals one sample (n = 1). Cardiac fibroblasts isolated from specific mouse lines are referred to by their descriptor, for example diabetic mice are referred to as diabetic fibroblasts.

### 2.4. Cell Culture and Treatment with Pharmacological Modifiers

Cells were passaged when 90–95% confluency was reached using a 0.25% trypsin/0.1% ethylenediaminetetraacetic acid (trypsin/EDTA) solution (Life Technology). All experiments were performed with passage 1 fibroblasts in order to ensure maintenance of cell in vivo phenotype within the in vitro system. Once fibroblasts reached 90–95% confluency, cells were rinsed with sterile 1X phosphate buffered saline solution (1× PBS), followed by incubating fibroblasts in starving DMEM (0.01% FBS) for 24 h. After 24 h, cell media was replaced with fresh low serum DMEM and incubated for 1 h. Next, pharmacological modifiers were added to the fibroblasts. The following modifiers were utilized within this study: EPAC (100 µM), exogenous AGEs (0.5 mg/mL; albumin, glycated human, Sigma Aldrich A8301), PKC-ζ pseudosubstrate (1 µg/mL; ps-PKC-ζ), Rap1a siRNA (100 nM), and scrambled control siRNA (100 nM). Cardiac fibroblasts and pharmacological modifiers were placed in incubator for 24 h. After 24 h the cells were collected for protein isolation and western blot analysis. Rap1a siRNA was utilized for the experimental study design in order to reduce Rap1a expression in cells that are able to produce functional Rap1a and allowed for examination of the impact temporal reduction of Rap1a has on cardiac fibroblast protein expression. Exposing cells with Rap1a to Rap1a siRNA also served as a secondary method to examine the impact of Rap1a on cardiac fibroblasts. Data for scrambled control siRNA is presented here 10.6084/m9.figshare.12625409.

### 2.5. Protein Isolation and Western Blot Analysis

Protein was isolated from cardiac fibroblasts using modified Hunter buffer (MHB: 75 mM NaCl, 0.5 mM orthovanadate, 5 mM Tris-Base, 0.5 mM ECTA, 0.5 mM EGTA, 1% Triton X-100, 0.25% NP-40, pH 7.4) and freshly added Halt protease inhibitor cocktail (100×; Thermo Fisher). Fibroblasts were incubated on ice with MHB for 10 min, followed by probe-sonication. Cell lysates were centrifuged for 15 min at 32,000× *g* at 4 °C and supernatant was removed and stored at −80 °C. Concentrations of protein were determined using a bicinchoninic acid assay (BCA; Pierce Biotechnology, city, abbreviation of state if USA, country) according to manufacturer’s directions; 10 µg of protein were loaded per sample for western blot analysis. Primary antibodies used were as follows: monoclonal alpha smooth muscle action (α-SMA, 42 kDa; 1:400; Sigma Aldrich #2547), phosphorylated nuclear factor kappa-light-chain-enhancer of activated B cells (p-NF-κB, 65 kDa; 1:400; Santa Cruz Biotechnology sc-136548), superoxide dismutase 1 (SOD-1, 23 kDa; 1:400; Santa Cruz Biotechnology sc-101523), repressor activator protein 1a (Rap1a, 21 kDa; 1:400; abcam ab96223), phosphorylated extracellular signal-regulated kinase (p-ERK1/2, 42 and 44kDa; 1:400; Santa Cruz Biotechnology sc-7383), extracellular signal-regulated kinase 1 and 2 (ERK1/2 44 and 42 kDa respectively; 1:400; Santa Cruz Biotechnology sc-271269 and sc-1647), phosphorylated protein kinase C zeta (p-PKC-ζ, 72 kDa; 1:400; Abcam ab62372), and beta tubulin (β-tubulin, 55 kDa; 1:400; Santa Cruz Biotechnology sc-398937). Brilliant Blue Coomassie staining was used to label total protein. β-tubulin was used as a loading control to normalize protein expression for all proteins except for p-ERK1/2. P-ERK1/2 was normalized to total ERK1/2 protein expression. β-tubulin was utilized as a loading control for p-PKC-ζ and p-NF-κB due to the antibodies used to assess total protein for PKC- ζ and NF-κB were not reliable on a consistent basis. Due to this limitation within our study design, we refer to the results generated from p-NF-κB and p-PKC-ζ as changes in protein expression and not as changes in activity. An Invitrogen iBRIGHT™ imaging system was used to visualize western blots, and Image J was used for analysis. Representative western blot images are depicted above each graph but are not presented as a continuous blot due to running order not aligning with the graphical order of the samples. The original western blot images are available at DOI:10.6084/m9.figshare.12625409.

### 2.6. Hydrogen Peroxide Assay

Protein lysates were used with OxiSelect hydrogen peroxide/peroxidase assay kit (BioLabs, STA-344) following manufacturer instructions, to assess concentration of hydrogen peroxide. Briefly, protein samples were incubated for 30 min with hydrogen peroxide working solution at room temperature in the dark. The colorimetric assay was analyzed with spectrometer at wavelength 540 nm. Concentration of hydrogen peroxide was determined based off a standard curve.

### 2.7. Statistical Analysis

**Figure 1:**
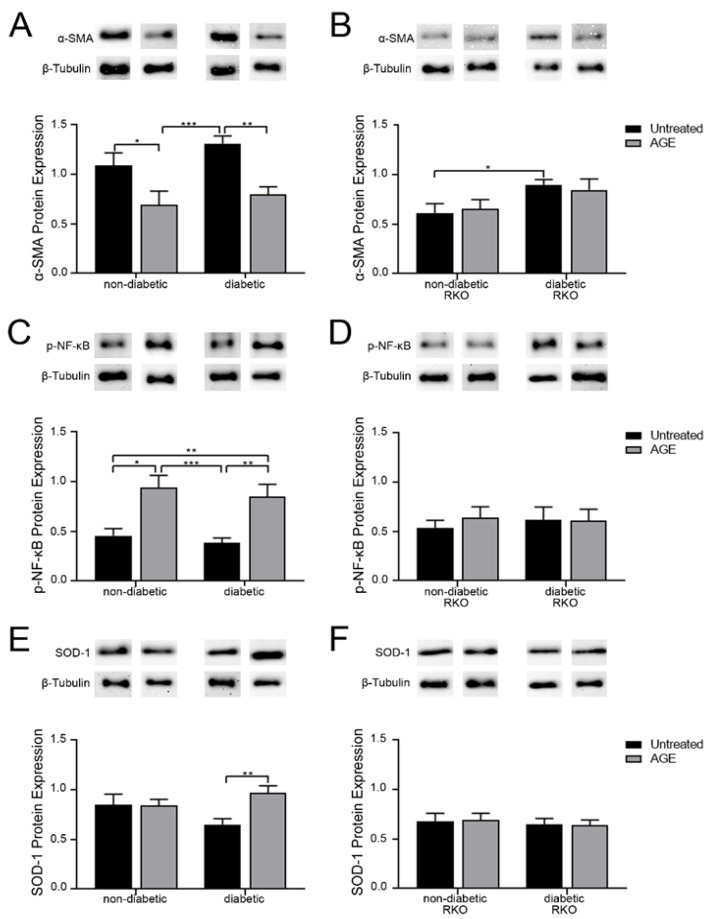
Diabetic conditions and exogenous AGEs caused a shift in expression of proteins associated with the RAGE signaling cascade. Cardiac fibroblasts were isolated from non-diabetic, diabetic, non-diabetic RKO, and diabetic RKO mice hearts and were either untreated or treated with exogenous AGEs (0.5 mg/mL). Protein expression was assessed for (**A,B**) α-SMA (42 kDA), (**C**,**D**) p-NF-κB (65 kDa), and (**E**,**F**) SOD-1 (23 kDa) in cardiac fibroblasts. Expression data were normalized to β-tubulin (55 kDa) protein expression and mean ± SEM are depicted on graph (n = 8–12). Representative western blot images are shown above graphs, but are not displayed as a continuous blot due to running order on the blot not aligning with graphical order of samples. However, original western blot images are available at DOI:10.6084/m9.figshare.12625409. A two-way ANOVA followed by a Fisher’s protected least significant difference post hoc determined significance. (* *p* < 0.05, ** *p* < 0.01, *** *p* < 0.001).

Graph Prism software, version 8.4.2 was used for statistical analysis. A two-way ANOVA was conducted to determine significance for the data presented in Figure 1. A one-way ANOVA was conducted for all figures except for Figure 1. A Fisher’s protected least significant difference post hoc test was conducted to determine differences between groups. Statistical p values indicated in the result section referred to the p value generated by the ANOVA and are not indicating the p value for the post hoc analysis. The p value for the post hoc analysis is indicated in the figures.

## 3. Results

### 3.1. Diabetic Conditions and Exogenous AGEs Caused a Shift in Expression of Proteins Associated with the RAGE Signaling Cascade

To assess the impact of RAGE signaling on cardiac fibroblasts, protein expression in untreated and AGE treated fibroblasts was examined. Protein expression analysis found that untreated non-diabetic and diabetic fibroblasts with functional RAGE had significantly more α-SMA expression compared to cells treated with exogenous AGEs (Figure 1A; two-way ANOVA, treatment *p* = 0.0002). Cardiac fibroblasts, both non-diabetic and diabetic, lacking functional RAGE (RAGE knockout; RKO) did not have a change in α-SMA expression between untreated and exogenous AGE treatment groups (Figure 1B; two-way ANOVA, treatment *p* = 0.9649). However, α-SMA expression was impacted by diabetic conditions in RKO cells where diabetic RKO fibroblasts had significantly more α-SMA compared to non-diabetic RKO (two-way ANOVA, genotype *p* = 0.0182). Exogenous AGE treatment resulted in significantly higher expression of p-NF-κB in non-diabetic and diabetic fibroblasts with functional RAGE, whereas p-NF-κB expression in RKO fibroblasts was not altered with exogenous AGE exposure (Figure 1C,D; two-way ANOVA treatment *p* < 0.0001 and *p* = 0.6929, respectively). Oxidative stress indicator, SOD-1 was significantly higher in diabetic cells treated with exogenous AGEs but not in non-diabetic cells (Figure 1E; two-way ANOVA, treatment *p* = 0.0522). RKO cells did not exhibit any changes in SOD-1 expression when treated with exogenous AGEs (Figure 1F; two-way ANOVA, treatment *p* = 0.9671). These results indicate AGE treatment decreased α-SMA and increased p-NF-κB expression in fibroblasts with functional RAGE.

### 3.2. Treatment with Exogenous AGEs and EPAC Resulted in an Increase in Rap1a Protein Expression

**Figure 2:**
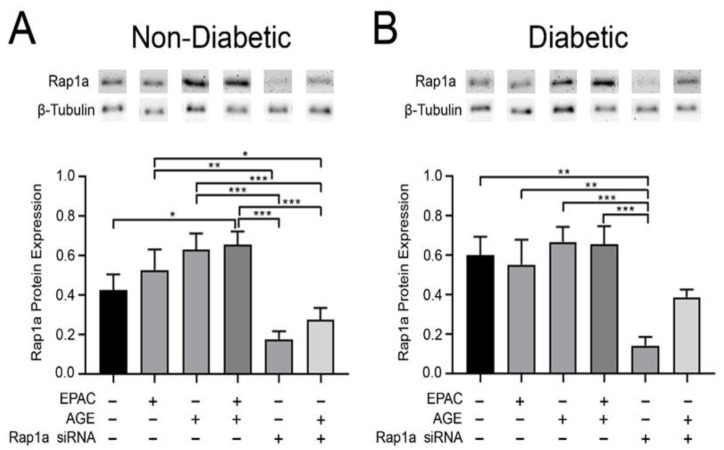
Treatment with exogenous AGEs and EPAC induced an increase in Rap1a protein expression. Cardiac fibroblasts were isolated from non-diabetic and diabetic mice hearts. Fibroblasts were either untreated, treated with a single pharmacological modifier, or treated in combination with EPAC (100 µM), EPAC+AGE (100 µM and 0.5 mg/mL, respectively), Rap1a siRNA (100 nM), or Rap1a siRNA + AGE (100 nM and 0.5 mg/mL, respectively). Rap1a (21 kDa) protein expression was assessed in (**A**) non-diabetic and (**B**) diabetic fibroblasts. Expression of Rap1a was normalized to β-tubulin (55 kDa) and mean ± SEM are depicted (n = 5–11). Representative western blot images are shown above graphs but are not displayed as a continuous blot due to running order on the blot not aligning with graphical order of samples. However, original western blot images are available at DOI:10.6084/m9.figshare.12625409. Statistical analysis consisted of one-way ANOVA followed by a Fisher’s protected least significant difference post hoc (* *p* < 0.5, ** *p* < 0.01, *** *p* < 0.001).

Examination of the relationship between Rap1a and the AGE/RAGE cascade was conducted by assessing the impact of AGE/RAGE signaling on Rap1a protein expression. Treatment of non-diabetic fibroblasts with exogenous AGEs and EPAC + exogenous AGEs caused an increase in Rap1a expression compared to untreated cells, but this change in Rap1a expression remained the same in diabetic cells (Figure 2A,B; one-way ANOVA *p* = 0.0002 and *p* = 0.0017, respectively). Treatment with Rap1a siRNA caused a significant decrease in Rap1a expression in non-diabetic and diabetic cells compared untreated cells. This decrease was attenuated, in both non-diabetic and diabetic cells, when cells were treated with Rap1a siRNA and exogenous AGEs. However, the expression of Rap1a in cells treated with Rap1a siRNA + exogenous AGEs did not reach the levels of Rap1a expression noted in untreated cells. The data indicates Rap1a expression can be modified by both exogenous AGEs and Rap1a siRNA.

### 3.3. Increased AGE/RAGE Signaling and Reduced Rap1a Expression Resulted in a Decrease in α-SMA Expression

**Figure 3:**
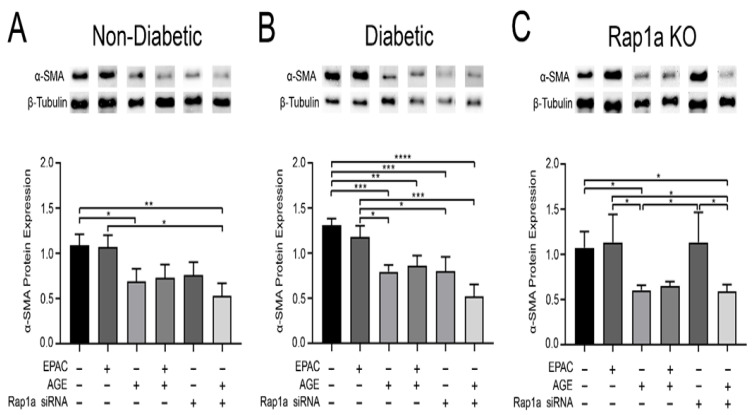
Increased AGE/RAGE signaling and reduced Rap1a expression induced a decrease in α-SMA expression. Cardiac fibroblasts were isolated from (**A**) non-diabetic, (**B**) diabetic, and (**C**) Rap1a KO mice hearts. Fibroblasts were either untreated, treated with a single pharmacological modifier, or treated in combination with EPAC (100 µM), EPAC + AGE (100 µM and 0.5 mg/mL, respectively), Rap1a siRNA (100 nM), or Rap1a siRNA + AGE (100 nM and 0.5 mg/mL, respectively). α-SMA (42 kDa) expression was assessed in cardiac fibroblasts. Expression of α-SMA was normalized to β-tubulin (55 kDa) and mean ± SEM are depicted on graphs (n = 5–11). Data depicted for exogenous AGE treatment group for non-diabetic and diabetic fibroblasts were previously presented Figure 1. Representative western blot images are shown above graphs but are not displayed as a continuous blot due to running order on the blot not aligning with graphical order of samples. However, original western blot images are available at DOI:10.6084/m9.figshare.12625409. Statistical analysis consisted of a one-way ANOVA followed by a Fisher’s protected least significant difference post hoc (* *p* < 0.5, ** *p* < 0.01, *** *p* < 0.001, **** *p* < 0.0001).

To determine the impact of AGE/RAGE signaling on fibroblast differentiation, changes in α-SMA expression were assessed in non-diabetic, diabetic, and Rap1a KO fibroblasts. Treatment with EPAC did not alter α-SMA expression in non-diabetic, diabetic, or Rap1a KO fibroblasts (Figure 3A–C). Treatment with exogenous AGEs caused significant decrease in α-SMA expression in non-diabetic, diabetic, and Rap1a KO fibroblasts (Figure 3A–C; one-way ANOVA, *p* = 0.0307, *p* < 0.0001, and *p* = 0.0362, respectively). While α-SMA expression in cells treated with exogenous AGEs + EPAC was less than in untreated cells (significantly less only in diabetic cells), it did not differ from cells treated solely with exogenous AGEs. Both non-diabetic and diabetic cells exhibited a decrease in α-SMA protein expression when treated with Rap1a siRNA; however, the decrease displayed by diabetic cells was significantly different compared to untreated diabetic cells. Rap1a KO cells did not exhibit any changes in α-SMA expression when treated with Rap1a siRNA. Treatment with Rap1a siRNA and exogenous AGEs resulted in significantly less α-SMA expression in non-diabetic, diabetic, and Rap1a KO cells. Both non-diabetic and diabetic RKO cardiac fibroblasts did not display any significant changes in α-SMA expression in the different treatment groups (Supplementary Figure S1A,B; one-way ANOVA *p* = 0.9639 and *p* = 0.3769, respectively). These results suggested that exogenous AGEs led to a decrease in α-SMA expression which further loss was prevented by the presence of Rap1a.

### 3.4. The Presence of Rap1a Mediated an AGE/RAGE Induced Increase in Inflammation Mediator p-NF-κB Expression

**Figure 4:**
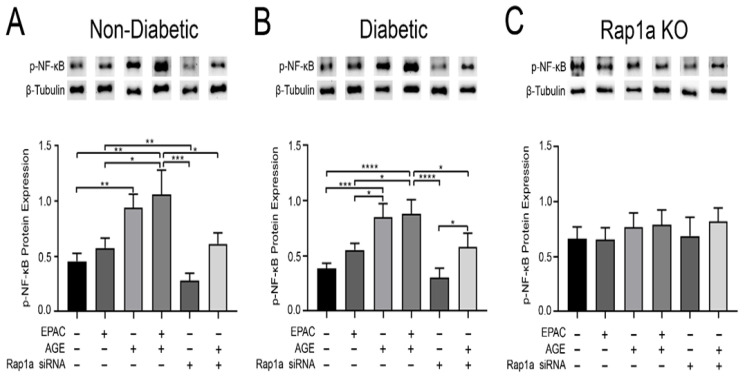
The presence of Rap1a mediated an AGE/RAGE induced increase in p-NF-κB expression. Cardiac fibroblasts isolated from (**A**) non-diabetic, (**B**) diabetic, and (**C**) Rap1a KO hearts were either untreated or treated with pharmacological modifiers, either solo or in combination: EPAC (100 µM), EPAC + AGE (100 µM and 0.5 mg/mL, respectively), Rap1a siRNA (100 nM), or Rap1a siRNA + AGE (100 nM and 0.5 mg/mL, respectively). Phosphorylation of NF-κB (65 kDa) was assessed and protein expression was normalized to β-tubulin (55 kDa) expression. Data depicted for exogenous AGE treatment group for non-diabetic and diabetic fibroblasts were previously presented Figure 1. Representative western blot images are shown above graphs but are not displayed as a continuous blot due to running order on the blot not aligning with graphical order of samples. However, original western blot images are available at DOI:10.6084/m9.figshare.12625409. Graph depicts mean ± SEM with n = 5–9 and statistical analysis consisted of one-way ANOVA followed by a Fisher’s protected least significant difference post hoc (* *p* < 0.05, ** *p* < 0.01, *** *p* < 0.001, **** *p* < 0.0001).

Inflammation is a well-described outcome of AGE/RAGE signaling and in order to determine the impact of Rap1a and AGE/RAGE signaling on inflammation, we examined changes in p-NF-κB expression. Treatment with EPAC alone did not cause a significant change in p-NF-κB expression while treatment with exogenous AGEs resulted in a significant increase in p-NF-κB expression in both non-diabetic and diabetic fibroblasts compared to untreated cells (Figure 4A,B; one-way ANOVA *p* = 0.0009 and *p* ≤ 0.0001, respectively). Furthermore, the combined exposure of exogenous AGE and EPAC caused significantly more p-NF-κB expression in non-diabetic and diabetic cells; however, expression of p-NF-κB was not greater than exogenous AGE treatment alone. Reduction of Rap1a expression via Rap1a siRNA did not produce a change in p-NF-κB in non-diabetic and diabetic cells compared to untreated cells. However, expression of p-NF-κB in siRNA treated non-diabetic and diabetic cells was significant less than exogenous AGE treated non-diabetic and diabetic fibroblasts. Non-diabetic and diabetic cells treated with exogenous AGEs + Rap1a siRNA displayed significantly less p-NF-κB expression compared to cells treated with exogenous AGE alone. Rap1a KO cells did not display any changes in p-NF-κB protein expression when exposure to the pharmacological modifiers (Figure 4C; one-way ANOVA *p* = 0.9134). Similarly, non-diabetic and diabetic RKO fibroblasts did not exhibit any changes in p-NF-κB expression between the different treatment groups (Supplementary Figure S1C,D; one-way ANOVA *p* = 0.9897 and *p* = 0.9936, respectively). The data indicates that exogenous AGEs with Rap1a produced an increase in p-NF-κB expression.

### 3.5. Reduced Rap1a Expression Caused Increased SOD-1 Expression

**Figure 5:**
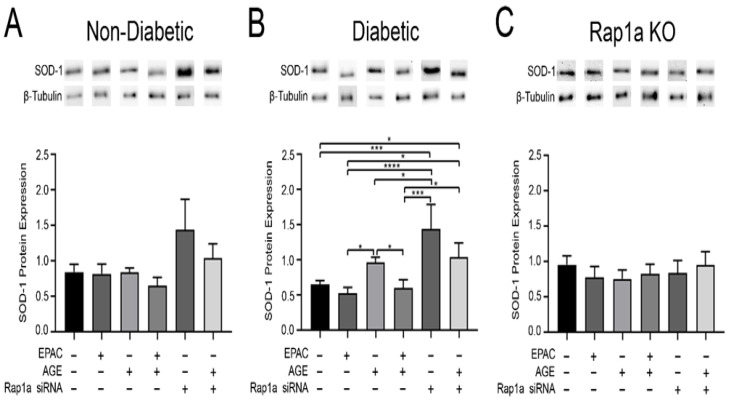
Reduced Rap1a expression caused increased SOD-1 expression. (**A**) Non-diabetic, (**B**) diabetic, and (**C**) Rap1a KO cardiac fibroblasts were isolated and treated with different combinations of EPAC (100 µM), AGE (0.5 mg/mL), and Rap1a siRNA (100 nM). SOD-1 (23 kDa) protein expression was normalized to β-tubulin (55 kDa) protein expression. Data depicted for exogenous AGE treatment group for non-diabetic and diabetic fibroblasts were previously presented Figure 1. Representative western blot images are shown above graphs but are not displayed as a continuous blot due to running order on the blot not aligning with graphical order of samples. However, original western blot images are available at DOI:10.6084/m9.figshare.12625409. Mean ± SEM are depicted in graphs (n = 7–9) with significance determined by one-way ANOVA followed by a Fisher’s protected least significant difference post hoc (* *p* < 0.05, *** *p* < 0.001, **** *p* < 0.0001).

Examination of SOD-1 expression was conducted to assess the effects of Rap1a activity and AGE/RAGE signaling have on mediating changes in proteins associated with oxidative stress within cardiac fibroblasts. Non-diabetic and diabetic fibroblasts did not exhibit any changes in SOD-1 expression when treated with EPAC, exogenous AGEs, or exogenous AGEs + EPAC compared to untreated controls (Figure 5A,B; one-way ANOVA *p* = 0.0868 and *p* = 0.0003, respectively). However, treatment with Rap1a siRNA resulted in an increase in SOD-1 expression in non-diabetic and diabetic fibroblasts, and there was a significant increase in SOD-1 expression in diabetic cells. Non-diabetic and diabetic fibroblasts treated with exogenous AGEs + Rap1a siRNA saw a slight increase in SOD-1 expression, but it was only significantly different in diabetic cells compared to untreated control. A similar pattern was noted in non-diabetic and diabetic RKO fibroblasts with regard to Rap1a siRNA treatment. RKO cells treated with Rap1a siRNA and exogenous AGEs + Rap1a siRNA displayed significantly more SOD-1 expression compared to untreated RKO cells (Supplementary Figure S1E,F; one-way ANOVA *p* = 0.0019 and *p* < 0.0001, respectively). Treatment groups that did not contain Rap1a siRNA showed no change in SOD-1 expression compared to the untreated control in both non-diabetic and diabetic RKO fibroblasts. In contrast, Rap1a KO fibroblasts did not exhibit any changes in SOD-1 expression when exposed to the different treatment groups (Figure 5C; one-way ANOVA *p* = 0.8942). The results indicate that SOD-1 expression was increased when Rap1a expression was reduced.

### 3.6. AGE/RAGE Signaling Induced Elevated Levels of Hydrogen Peroxide in Non-Diabetic and Rap1a KO Cardiac Fibroblasts

**Figure 6:**
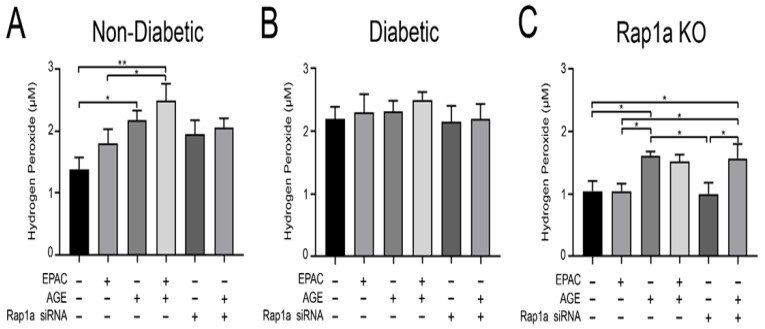
AGE/RAGE signaling induced elevated levels of hydrogen peroxide in non-diabetic and Rap1a KO cardiac fibroblasts. Cardiac fibroblasts isolated from (**A**) non-diabetic, (**B**) diabetic, and (**C**) Rap1a KO mice were treated with different combinations of pharmacological modifiers: EPAC (100 µM), exogenous AGEs (0.5 mg/mL), and Rap1a siRNA (100 nM). Cell lysates were collected and assessed for concentration of hydrogen peroxide. Values graphed are mean ± SEM (n = 4–13) and a one-way ANOVA followed by a Fisher’s protected least significant difference post hoc was conducted to determine significance (* *p* < 0.05 and ** *p* < 0.01).

Due to the impact AGE/RAGE signaling on the expression of proteins associated with inflammation/oxidative stress, hydrogen peroxide concentration was assessed in cardiac fibroblasts. Non-diabetic fibroblasts treated with exogenous AGEs as well as exogenous AGEs + EPAC displayed a significant increase in hydrogen peroxide concentration compared to untreated cells (Figure 6A; one-way ANOVA *p* = 0.0430). While exposure to Rap1a siRNA did not cause a significant change in hydrogen peroxide concentration, the levels of hydrogen peroxide were higher than untreated cells but lower than exogenous AGE+EPAC treated cell levels. Diabetic fibroblasts did not exhibit any changes in hydrogen peroxide concentration between the different treatment groups (Figure 6B; one-way ANOVA *p* = 0.9290). Treatment groups containing exogenous AGEs produced a significant increase in hydrogen peroxide concentration in Rap1a KO fibroblasts (Figure 6C; one-way ANOVA *p* = 0.0204). Non-diabetic and diabetic RKO fibroblasts did not show any changes in hydrogen peroxide levels between the different treatment groups and the untreated cells (Supplementary Figure S2A,B; one-way ANOVA *p* = 0.3684 and *p* = 0.9980, respectively). In addition, diabetic fibroblasts displayed significantly more hydrogen peroxide concentration compared to non-diabetic, Rap1a KO, non-diabetic RKO, and diabetic RKO cells (Supplementary Figure S3; one-way ANOVA *p* = 0.0012). The data suggests that increased AGE activation of RAGE produced an increase in hydrogen peroxide concentration in non-diabetic and Rap1a KO fibroblasts.

### 3.7. Rap1a Mediated AGE/RAGE Induced ERK1/2 Activation in Cardiac Fibroblasts

**Figure 7:**
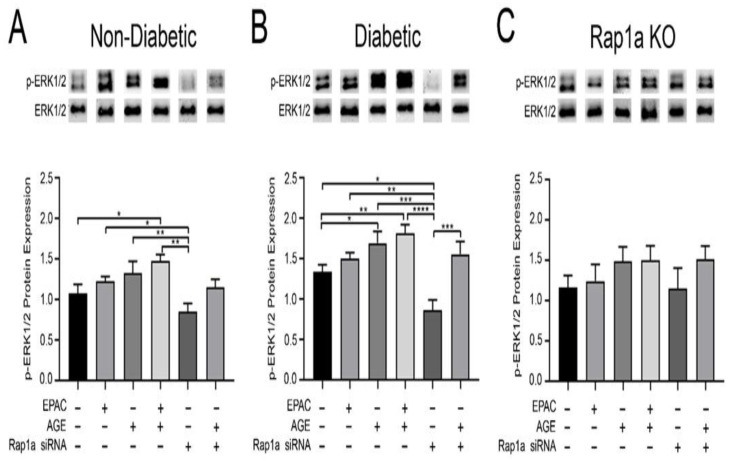
Rap1a mediated AGE/RAGE induced ERK1/2 activation in cardiac fibroblasts. Cardiac fibroblasts isolated from (**A**) non-diabetic, (**B**) diabetic, and (**C**) Rap1a KO mouse hearts were treated with pharmacological modifiers either solo or in combination: EPAC (100 µM), exogenous AGEs (0.5 mg/mL), and Rap1a siRNA (100 nM). Protein expression of p-ERK1/2 (42 and 44 kDa) was normalized to total ERK1/2 (44 and 42 kDa, respectively) expression. Representative western blot images are shown above graphs but are not displayed as a continuous blot due to running order on the blot not aligning with graphical order of samples. However, original western blot images are available at DOI:10.6084/m9.figshare.12625409. Values graphed represent mean ± SEM (n = 6–10) with an one-way ANOVA followed by a Fisher’s protected least significant difference post hoc determining significance (* *p* < 0.05, ** *p* < 0.01, *** *p* < 0.001, **** *p* < 0.0001).

In order to assess downstream AGE/RAGE signaling proteins, the expression of p-ERK1/2 was examined. EPAC treated non-diabetic and diabetic fibroblasts did not have significant changes in p-ERK1/2 expression compared to untreated cells (Figure 7A,B). Treatment with exogenous AGEs caused an increase in p-ERK1/2 expression in non-diabetic cells, and a significant increase in diabetic fibroblasts compared to untreated control (Figure 7A,B; one-way ANOVA *p* = 0.0061 and *p* = 0.0004, respectively). Rap1a KO fibroblasts did not exhibit an increase in p-ERK expression with EPAC treatment but did show a slight yet non-significant change when exposed to exogenous AGEs when compared to Rap1a untreated cells (Figure 7C; one-way ANOVA *p* = 0.8755). Treatment with exogenous AGEs + EPAC produced a significant increase in p-ERK1/2 in non-diabetic and diabetic fibroblasts and no change in Rap1a KO cells. Rap1a siRNA treatment caused a significant decrease in p-ERK1/2 expression compared to exogenous AGE treated cells in both non-diabetic and diabetic cells. However, expression of p-ERK1/2 in Rap1a siRNA treated non-diabetic and diabetic cells did not significantly differ from the untreated cells. Rap1a KO cells showed no change in expression levels of p-ERK1/2 in untreated and Rap1a siRNA treated groups. The decrease in p-ERK1/2 expression was attenuated in both non-diabetic and diabetic fibroblasts with combined exposure of both exogenous AGEs and Rap1a siRNA. The level of p-ERK1/2 expression was not restored to AGE or AGE+EPAC levels. Non-diabetic RKO cells did not display any changes in p-ERK1/2 expression within any of the different treatment groups (Supplementary Figure S4A; one-way ANOVA *p* = 0.9808). However, some changes in p-ERK1/2 expression did occur with diabetic RKO fibroblasts when Rap1a activity was altered where treatment with Rap1a siRNA to cause a significant decrease in p-ERK1/2 expression compared to untreated cells (Supplementary Figure S4B; one-way ANOVA *p* = 0.0025). These results suggest that exogenous AGEs with EPAC activation of Rap1a led to an increase in ERK1/2 activity in fibroblasts with functional RAGE and Rap1a.

### 3.8. Rap1a Modified p-PKC-ζ Expression Both Dependently and Independently of AGE/RAGE Signaling

**Figure 8:**
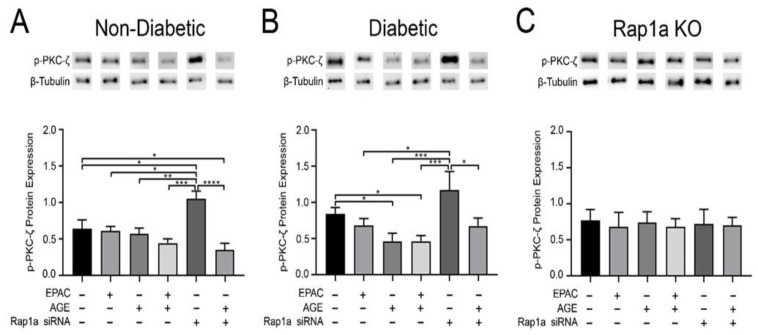
Rap1a modified p-PKC-ζ expression both dependently and independently of AGE/RAGE signaling. Total protein was isolated from (**A**) non-diabetic, (**B**) diabetic, and (**C**) Rap1a KO cardiac fibroblasts. Pharmacological modifiers EPAC (100 µM), exogenous AGEs (0.5 mg/mL), and Rap1a siRNA (100 nM) were added to fibroblasts either solo or in combination. p-PKC-ζ (72 kDa) protein expression was normalized to β-tubulin (55 kDa) protein expression. Representative western blot images are shown above graphs but are not displayed as a continuous blot due to running order on the blot not aligning with graphical order of samples. However, original western blot images are available at DOI:10.6084/m9.figshare.12625409. Mean ± SEM are displayed in the graphs (n = 5–10) and significance was determined with a one-way ANOVA followed by a Fisher’s protected least significant difference post hoc (* *p* < 0.05, ** *p* < 0.01, *** *p* < 0.001, **** *p* < 0.0001).

In order to better understand the AGE/RAGE signaling cascade and the impact of Rap1a, we examined changes in p-PKC-ζ expression. Non-diabetic fibroblasts treated with EPAC or exogenous AGEs did not display any significant changes in p-PKC-ζ expression (Figure 8A; one-way ANOVA *p* = 0.0021). However, reduction in Rap1a expression through Rap1a siRNA resulted in a significant increase in p-PKC-ζ expression in non-diabetic cells compared to untreated non-diabetic fibroblasts. The increase in p-PKC-ζ was not exhibited when non-diabetic cells were treated with Rap1a siRNA + exogenous AGEs. The same trend occurred with diabetic fibroblasts with slight modifications. Diabetic cells treated with exogenous AGEs as well as EPAC + exogenous AGEs exhibited a significant decrease in p-PKC-ζ expression compared to untreated diabetic cells (Figure 8B; one-way ANOVA *p* = 0.0052), while treatment with Rap1a siRNA caused significantly more p-PKC-ζ expression to occur compared to EPAC, exogenous AGE, and EPAC + exogenous AGE treated cells. In contrast, Rap1a KO fibroblasts did not show any changes in p-PKC-ζ expression within the different treatment groups (Figure 8C; one-way ANOVA *p* = 0.9982). Both non-diabetic and diabetic RKO cardiac fibroblasts did not exhibit any changes in p-PKC-ζ expression within the different treatment groups (Supplementary Figure S4C,D; one-way ANOVA *p* = 0.9380 and *p* = 0.8907, respectively). The data indicates that reduced Rap1a expression resulted in a significant increase in p-PKC- expression in fibroblasts with functional RAGE and Rap1a.

### 3.9. Inhibition of p-PKC-ζ Caused a Slight Decrease in RAGE Associated Proteins Which Was Further Attenuated with Rap1a siRNA Treatment

**Figure 9:**
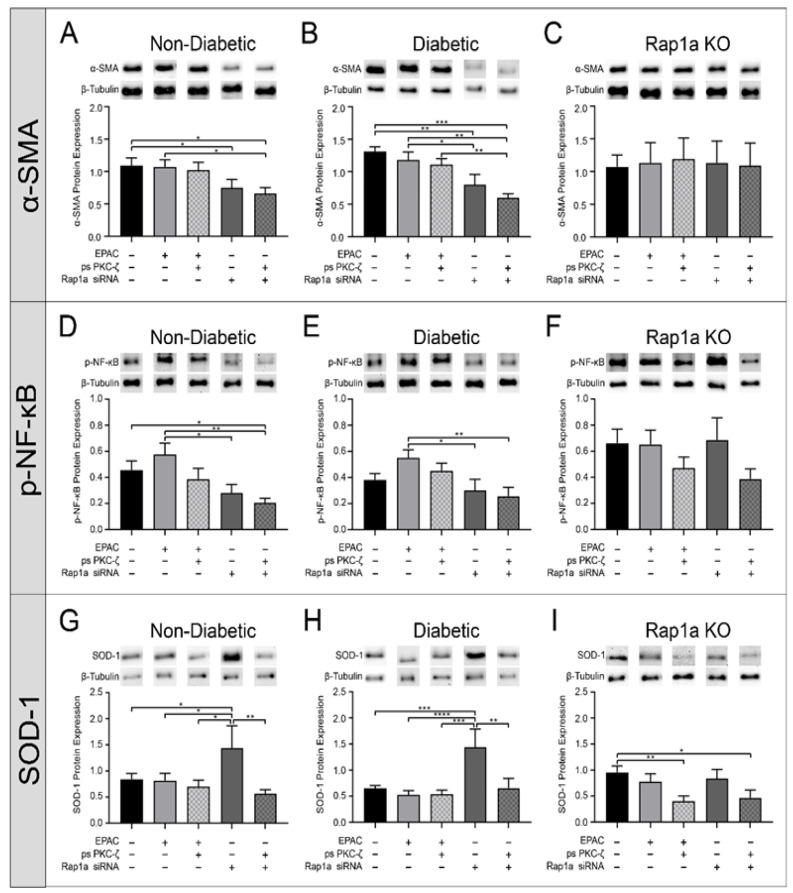
Inhibition of p-PKC-ζ caused a decreased in RAGE associated proteins which was further attenuated with Rap1a siRNA treatment. Cardiac fibroblasts were isolated from non-diabetic, diabetic, and Rap1a KO mice. Cells were treated either with EPAC (100 µM), EPAC + ps PKC-ζ (100 µM and 1 µg/mL, respectively), Rap1a siRNA (100 nM), or Rap1a siRNA + ps PKC-ζ (100 nM and 1 µg/mL, respectively), followed by collection of total protein. (**A**–**C**) α-SMA (42 kDa), (**D**–**F**) p-NF-κB (65 kDa), and (**G**–**I**) SOD-1 (23 kDa) protein expression was assessed. Protein expression was normalized to β-tubulin (55 kDa) protein expression and mean ± SEM were depicted in graph (n = 6–9). Representative western blot images are shown above graphs but are not displayed as a continuous blot due to running order on the blot not aligning with graphical order of samples. However, original western blot images are available at DOI:10.6084/m9.figshare.12625409. Data depicted for EPAC and Rap1a siRNA treatment groups were previously presented Figure 3, Figure 4 and Figure 5. Significance was assessed using a one-way ANOVA followed by a Fisher’s protected least significant difference post hoc (* *p* < 0.05, ** *p* < 0.01, *** *p* < 0.001, **** *p* < 0.0001).

Determining the intersection of Rap1a on the AGE/RAGE signaling cascade was accomplished by treating cardiac fibroblasts with ps PKC-ζ in the presence of increased or decreased Rap1a activity/expression. EPAC treatment did not cause a change in α-SMA expression in non-diabetic, diabetic, or Rap1a KO cardiac fibroblasts compared to untreated cells (Figure 9A–C; the one-way ANOVA indicated significance [non-diabetic *p* = 0.0454, diabetic *p* < 0.0001, and Rap1a KO *p* = 0.9988]; however, the post hoc analysis did not). Treatment with EPAC + ps PKC-ζ did not cause a change in α-SMA expression compared to untreated treated cells in all cell types except for Rap1a KO fibroblasts. Non-diabetic and diabetic cells exposed to Rap1a siRNA displayed a significant decrease in α-SMA expression, which was further attenuated with Rap1a siRNA + ps PKC-ζ treatment. Rap1a KO cells did not display a decrease in α-SMA expression with Rap1a siRNA or Rap1a siRNA + ps PKC-ζ treatments (Figure 9C).

A similar pattern occurred with p-NF-κB protein expression in isolated cardiac fibroblasts (Figure 9D–F). Non-diabetic and diabetic cells treated with EPAC exhibited a slight non-significant change in p-NF-κB expression compared to untreated cells, which did not occur with combined treatment of EPAC + ps PKC-ζ (Figure 9D,E; the one-way ANOVA indicated significance [*p* = 0.0224 and *p* = 0.0444, respectively] however, the post hoc analysis did not). Rap1a siRNA and Rap1a siRNA + ps PKC-ζ exposure caused a significant decrease in p-NF-κB expression in non-diabetic and diabetic fibroblasts compared to EPAC treated cells and a non-significant change compared to untreated cells. Rap1a KO fibroblasts only displayed a non-significant increase in p-NF-κB expression when exposed to treatment groups containing ps PKC-ζ (Figure 9F; one-way ANOVA *p* = 0.4748).

Changes in SOD-1 expression were affected by decreasing Rap1a and/or PKC-ζ activity. Exposure to EPAC treatment did not cause a change in SOD-1 expression in non-diabetic, diabetic, or Rap1a KO fibroblasts compared to untreated cells (Figure 9G–I; the one-way ANOVA indicated significance [non-diabetic *p* = 0.0452, diabetic *p* = 0.0010, and Rap1a KO *p* = 0.0348] however, the post hoc analysis did not). While SOD-1 expression did not respond to treatment with EPAC + ps PKC-ζ in non-diabetic and diabetic cells; Rap1a KO cells did show a significant decrease in SOD-1 expression compared to untreated cells. Non-diabetic and diabetic fibroblasts treated with Rap1a siRNA exhibited a significant increase in SOD-1 expression compared to untreated cells, this same pattern was not noted in Rap1a KO cells. Lastly, the combined treatment of Rap1a siRNA + ps PKC-ζ caused a significant decrease in SOD-1 expression only in Rap1a KO fibroblasts. This data suggests that Rap1a may impact AGE/RAGE signaling at the point of PKC-ζ.

## 4. Discussion

The objective of this study was to assess the impact of Rap1a activity on downstream AGE/RAGE signaling outcomes in cardiac fibroblasts, and the role of Rap1a as a potential modulator in the AGE/RAGE signaling cascade in diabetic cardiovascular complications. We hypothesized that Rap1a crosses the AGE/RAGE cascade to alter expression of AGE/RAGE associated signaling proteins in cardiac fibroblasts in type 2 diabetic mice. The results of this study indicated Rap1a crosses the AGE/RAGE signaling cascade and caused changes in signaling protein expression known to be associated with increased RAGE activation. Rap1a appeared to attenuate α-SMA expression, even with increased exogenous AGE exposure. Additionally, when cells expressed Rap1a in combination with increased AGE/RAGE signaling it resulted in an increase in p-NF-κB expression, which correlated with an increase in hydrogen peroxide concentration, an indicator of possible oxidative stress. Lastly, we observed that the presence of Rap1a was able to reduce SOD-1 expression independently of AGE/RAGE signaling.

Individuals with diabetes have an increased risk for heart failure due to cardiac hypertrophy, which results in part to increased ECM remodeling by myofibroblasts. Studies have shown that increased ECM remodeling correlates with elevated levels of AGEs and α-SMA expression in cardiac fibroblasts [5,28,45]. α-SMA has been used extensively in research to assess both the myofibroblast population and possible ECM remodeling that occurs with cardiac fibrosis [46,47,48]. While AGE activation of RAGE has been shown to directly stimulate α-SMA expression, little research has been conducted to examine the impact modulators have on the AGE/RAGE cascade. Our findings demonstrated that acute exogenous AGE exposure caused a decrease in α-SMA expression, and this decrease was further extended when Rap1a expression was reduced by siRNA knockdown. These results suggested Rap1a may maintain or promote α-SMA expression in cardiac fibroblasts because when Rap1a expression is reduced, via siRNA, there was a decrease in expression. Furthermore, when cells were treated with exogenous AGEs and Rap1a siRNA, there was a greater decrease in α-SMA expression compared to when fibroblasts were treated solely with exogenous AGEs. Therefore, it appears that Rap1a helps promote α-SMA even when expression is being reduced by exogenous AGE treatment. Initially, it appeared the data presented in this study conflicted with findings previously published in the literature by demonstrating AGE treatment caused α-SMA expression to be reduced. A study by Simard et al. 2015 showed aortic vascular smooth muscle cells treated for 24 h with AGEs led to a decrease in α-SMA protein expression, whereas longer AGE exposure resulted in an increase in α-SMA expression [49]. Similar results have been noted in other studies that showed acute versus chronic AGE exposure can have different impacts on α-SMA expression in fibroblasts [2,45]. Therefore, it is important to investigate this relationship further. The data presented within this manuscript and in combination with data in the literature and previously published by our lab suggested that the length of AGE exposure, whether acute (hours) versus chronic (days to weeks) could explain the contradictory findings in this study regarding α-SMA expression. Our study provides an initial insight into the impact of exogenous AGEs and Rap1a on α-SMA expression and the possible implications these changes could have on ECM remodeling in cardiac fibroblasts. However, additional studies are necessary to determine the direct impact changes in α-SMA have on proteins associated with ECM remodeling. Overall, it appeared that acute AGE exposure shifted the RAGE signaling cascade away from promoting α-SMA, ECM remodeling indicator, expression and towards other downstream signaling proteins normally associated with inflammation and oxidative stress.

To determine the point of crossing of Rap1a and the AGE/RAGE cascade, changes in expression of RAGE-associated signaling proteins were assessed when PKC-ζ phosphorylation was inhibited with pseudosubstrate PKC. It was noted that inhibition of p-PKC-ζ combined with Rap1a silencing resulted in a greater loss of α-SMA expression. These findings could indicate either PKC-ζ inhibition decreased AGE/RAGE signaling outcomes, which led to either a reduction of α-SMA expression, or p-PKC-ζ inhibition blocked Rap1a from stimulating α-SMA expression. Our findings from Rap1a KO cells lends credence to the latter theory due to the lack of change in these cells. Conversely, should p-PKC-ζ inhibition have prevented AGE/RAGE signaling, there would be a predicted lack of change in α-SMA expression. Since a decrease in α-SMA protein expression was observed in Rap1a siRNA + ps PKC-ζ treated cells, it would appear that inhibition of p-PKC-ζ had a greater impact on preventing Rap1a activity than the AGE/RAGE signaling cascade. A similar pattern occurred when examining changes in p-NF-κB and SOD-1 expression with inhibition of PKC-ζ phosphorylation. These results indicated that Rap1a and PKC-ζ were both involved with regulating p-NF-κB, and SOD-1 expression. PKC-ζ could possibly act as a point of intersection for Rap1a to modify the AGE/RAGE signaling cascade. Previously, it has been demonstrated that Rap1a can influence PKC activity, which provides further support for the results presented within this manuscript [50]. However, additional studies would need to be conducted to provide additional support for this idea.

Acute AGE exposure appeared to decrease myofibroblast marker α-SMA expression, yet at the same time an increase in p-NF-κB expression, a signaling protein associated with inflammation and oxidative stress, was observed. Cardiac fibroblasts treated with exogenous AGEs had increased p-NF-κB expression, and protein expression was further increased when Rap1a was activated by EPAC stimulation. Elevated p-NF-κB expression was diminished when fibroblasts were treated with exogenous AGEs+Rap1a siRNA. Rap1a siRNA treatment alone reduced p-NF-κB levels to levels lower than untreated cells. These results, in combination with those observed in RKO fibroblasts in which no changes in p-NF-κB expression occurred, suggested Rap1a may be involved with AGE-mediated increases in NF-κB protein expression. Our results corresponded to previously conducted studies demonstrating treatment with exogenous AGEs triggered a RAGE specific increase in p-NF-κB expression [15,26,51]. In addition to AGEs, increased Rap1a activity has also been shown to increase p-NF-κB activity in a variety of cell types, such as mesenchymal stem cells, hepatocytes, and neutrophils [52,53]. This data provided further evidence that Rap1a possibly played a role in the AGE/RAGE signaling pathway by redirecting acute changes in AGE-exposed fibroblasts to increased p-NF-κB expression. Furthermore, the impact of Rap1a and AGE/RAGE mediated increased p-NF-κB could suggest Rap1a’s involvement in promoting RAGE mediated inflammation and oxidative stress. Multiple studies have shown RAGE activation utilizes NF-κB for inflammatory and oxidative stress responses [9,20,54]. However, due to the complex role NF-κB plays within the AGE/RAGE signaling cascade, it was necessary to further examine the function of NF-κB in mediating either RAGE induced inflammation and/or oxidative stress.

Increases in p-NF-κB could indicate the cells are undergoing either an inflammatory response or an increase in oxidative stress. Due to limitations within our study design, we were only able to examine changes in oxidative stress by measuring the concentration of hydrogen peroxide. These results showed changes in hydrogen peroxide concentration between the different treatment groups, which mirrored the changes observed in p-NF-κB protein expression. Therefore, indicating AGE/RAGE signaling may have utilized the NF-κB pathway to increase oxidative stress within the cardiac fibroblasts. Studies have shown increased AGE/RAGE signaling can cause increased NF-κB activity resulting in increased cytokine levels, such as raised TNF-α expression, which has been documented to induce elevated free radical (O_2_^−^) production [15,55,56]. Furthermore, a positive feedback loop exists that can upregulate both RAGE and TNF-α expression as a result of increased NF-κB activity [15,22]. Based on these studies, the increase we noted in p-NF-κB expression and hydrogen peroxide was most likely due to a combinatorial effect whereby each component promoted an increase in the other. Overall, the results suggested that AGE/RAGE signaling and Rap1a triggered an increase in oxidative stress, and NF-κB expression was possibly involved in this change.

The transcription factors, ERK1/2, participate in numerous intracellular signaling cascades, and they have been linked to the RAGE signaling cascade [24,57]. Therefore, p-ERK1/2 expression was assessed to determine if AGE activation of RAGE utilized ERK1/2 as an intermediate signaling molecule to increase p-NF-κB expression. It was found that p-ERK1/2 protein expression mirrored changes noted in p-NF-κB expression with each of the different treatment groups. These results indicated ERK1/2 may act downstream of AGE-mediated RAGE activation to induce the observed acute changes in p-NF-κB expression. Furthermore, we were able to delineate that Rap1a may act upstream of ERK1/2 to elicit changes in expression of p-ERK1/2, which possibly promoted p-NF-κB expression. These findings were supported by decreased p-ERK1/2 and p-NF-κB expression when a global knockout and a cell-specific knockdown of Rap1a expression occurred. This proposed mechanism aligns with data documented in the literature as it has been shown activation of RAGE led to increased p-ERK1/2 expression resulting in increased NF-κB activation [24,57]. A similar outcome was noted in vascular smooth muscle cells when cultured in hyperglycemic conditons [58]. Additional evidence showed Rap1 can produce an increase in p-ERK expression [59,60]. Dorn et al. 2012 showed similar results where Rap1 caused an increase in p-ERK1/2 expression, which prompted increased pro-inflammatory cytokine production [61]. This study also noted that Rap1 may not directly activate ERK1/2, but Rap1 may use another mediator such as Raf to initiate downstream changes [61]. Our study when combined with data presented in the literature indicated Rap1a may affect AGE/RAGE mediated increased ERK1/2 activity, it is worth noting that future studies are needed to determine the sequential cascade. Overall, our findings suggest that AGE activation of RAGE stimulates p-ERK1/2 to promote increased p-NF-κB expression and Rap1a stimulus by EPAC further exacerbated this outcome.

Rap1a is not the only factor capable of exerting influence on the AGE/RAGE signaling cascade. In this study, cells under diabetic conditions exhibited either a higher degree of protein expression and/or change in protein expression, specifically Rap1a, α-SMA, p-NF-κB, SOD-1, p-ERK1/2, and p-PKC-ζ as compared to untreated cells. These larger changes could be a result of increased basal levels of RAGE expression as noted in diabetics [5,62,63]. Pre-existing elevated levels of RAGE could prime the cells and allow for faster, greater, and more sustainable changes in protein expression as compared to their non-diabetic counterparts. However, there are specific proteins within cardiac fibroblasts that exhibit changes in expression due to diabetic conditions and are not solely impacted by AGE/RAGE signaling. For example, significant changes in p-ERK1/2 expression occurred within RKO diabetic cells while no changes were noted in non-diabetic RKO cells. Therefore, it would be reasonable to suggest that differences in p-ERK1/2 expression within diabetic RKO was due to diabetic conditions. Previous studies have shown multiple instances in which diabetic conditions and not RAGE signaling increased p-ERK1/2 expression [14,64]. Furthermore, it has been shown that Rap1 can influence p-ERK1/2 expression via activation of angiotensin II type 1 receptor (AT1R), which has been documented as a major observance in hearts from diabetic patients [65,66]. These studies provided a possible explanation for the changes in p-ERK1/2 expression within diabetic RKO fibroblast. Overall, it appeared that diabetic conditions could prime cardiac fibroblasts to respond to AGE/RAGE signaling more readily and result in acute changes.

The expression of RAGE signaling proteins have been shown to effect as well as be affected by increased oxidative stress [17,61]. Oxidative stress can be regulated by the expression of SOD which are involved with converting harmful superoxide radicals into hydrogen peroxide [67]. According to literature, exogenous AGEs can induce an increase in ROS, which in turn, increases SOD expression [14,26]. In contrast, increased RAGE activation has also been demonstrated to lead to SOD-2 inhibition in angiogenic progenitor cells via miR-21 [68]. In our study, we found exogenous AGE treatment did not change SOD-1 expression despite an increase in hydrogen peroxide production.

These findings suggested either RAGE activation prevented changes in SOD-1 expression, or RAGE signaling did not directly impact SOD-1 expression in cardiac fibroblasts. Considering the lack of change in SOD-1 expression in RKO fibroblasts, our results lend support to the latter idea. In that, acute exogenous AGE exposure in cardiac fibroblasts failed to impact SOD-1 protein expression. Further examination of the data showed SOD-1 expression increased when Rap1a expression was reduced; however, this increase in SOD-1 expression did not correlate with an overall change in hydrogen peroxide concentrations. While it would be anticipated that hydrogen peroxide levels would decrease with Rap1a silencing, this may not have occurred due to active RAGE signaling within these cells promoting hydrogen peroxide production via activation of NF-κB. This idea was supported by the lack of increased hydrogen peroxide in non-diabetic and diabetic RKO fibroblasts.

Rap1a has also been documented to modify oxidative stress through regulation of NADPH oxidase activity. Rap1a will co-localize, co-translocate, and co-immunoprecipitate with the NADPH p22^PHOX^ subunit [35,69]. Furthermore, reduction of Rap1a in macrophages resulted in slower production of superoxides [44]. Combining both literature findings and data presented in this study, there is a correlative trend that indicates Rap1a affected SOD-1 expression and hydrogen peroxide concentration by altering NADPH oxidase activity. To determine if Rap1a impacted SOD-1 expression either independently or in association with RAGE cascade signaling proteins, we examined p-PKC-ζ expression as it relates to AGE/RAGE signaling and its role in assembly of NADPH oxidase [19,20,70]. PKC-ζ phosphorylates the NADPH p47^PHOX^ subunit, leading to translocation of the NADPH cytosolic unit to the NADPH plasma membrane unit [70]. Assembly of NADPH oxidase allows for the generation of superoxide radicals, which contributes to production of oxidative stress [71,72]. We found that p-PKC-ζ expression was increased when cells were treated with Rap1a siRNA and the increase of p-PKC-ζ corresponded with higher levels of SOD-1 expression in cardiac fibroblasts. Therefore, it appeared when Rap1a levels were elevated it caused a preventative increase in p-PKC-ζ expression, which in turn led to lower levels of SOD-1. These results suggest Rap1a may influence PKC-ζ regulation of SOD-1 by altering activity of NADPH oxidase. A study by Fontayne et al., 2002 indicated that p47^PHOX^ interacted with p22^PHOX^ only when p47^PHOX^ was phosphorylated by a PKC isoform [70]. Therefore, Rap1a interacting with p22^PHOX^ could be the mechanism by which Rap1a regulates PKC-ζ effects on NADPH oxidase, and in turn, reduced SOD-1 expression [35]. While initial data suggested that this was a possible mechanism, additional studies would need to be conducted to determine if this trend continues.

**Figure 10:**
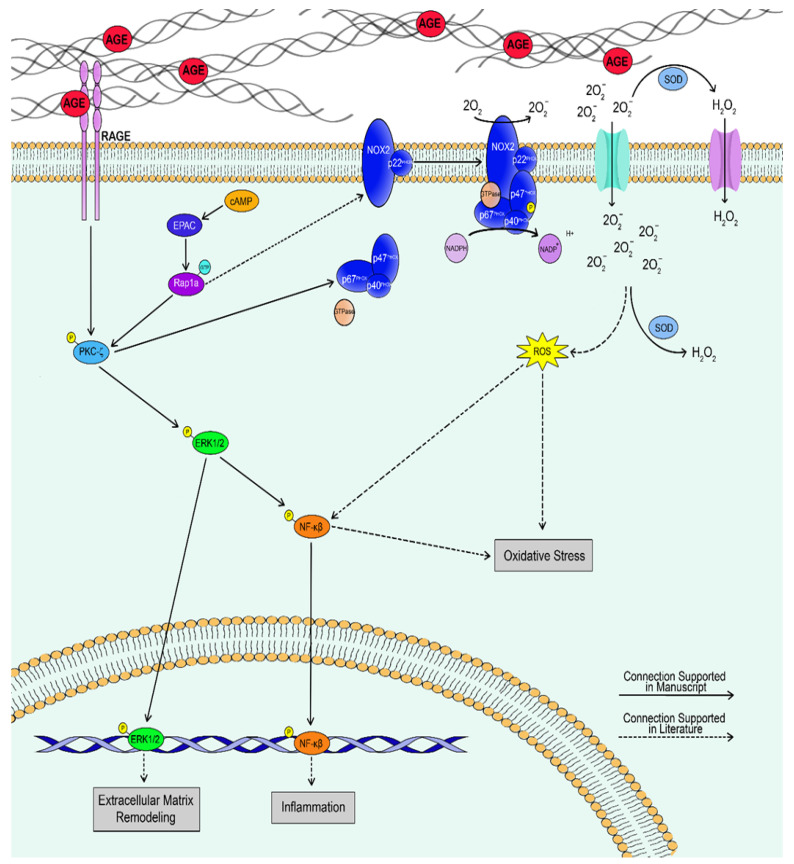
Diagram depicting the relationship between Rap1a and the AGE/RAGE signaling cascade. Potential interactions between proteins are shown with arrows with directionality of impact being denoted with arrow orientation. Solid line arrows indicate connections that are supported by evidence presented within this manuscript, while dash line arrows show connections that are supported within the literature.

The aim of this study was to provide initial insight into the impact of Rap1a on AGE/RAGE signaling proteins in cardiac fibroblasts. In summary, it appears that Rap1a possibly interacts with the AGE/RAGE signaling cascade to alter protein expression in cardiac fibroblasts. Acute exogenous AGE exposure in cardiac fibroblasts caused a decrease in α-SMA expression while inducing an increase in p-NF-κB expression. Within this paradigm, Rap1a appears to promote an AGE/RAGE-mediated increase in p-NF-κB expression as well as possibly maintain a basal level of expression of α-SMA in cardiac fibroblasts. These results suggest that Rap1a in combination with acute AGE exposure shifts cardiac fibroblasts away from myofibroblast differentiation and towards an oxidative stress response. However, more in-depth analysis would need to be conducted to determine if this was the case. The point of intersection of Rap1a to the AGE/RAGE cascade appears to be at the level of PKC-ζ. Rap1a may utilize PKC-ζ to influence downstream targets (α-SMA, p-ERK1/2, and p-NF-κB), as well as affect the ability of PKC-ζ to promote SOD-1 expression. Figure 10 provides a visual representation of the effects of Rap1a and AGE/RAGE signaling on cardiac fibroblasts. The data presented within this manuscript highlight the impact of Rap1a with acute AGE exposure on RAGE signaling proteins but does not address the impact of chronic AGE exposure, which is a characteristic of those with diabetes. Further studies will need to be conducted to assess how Rap1a with chronic AGE exposure will affect the AGE/RAGE signaling proteins in cardiac fibroblasts. This initial study provides, for the first time, the effect that Rap1a has on regulating the expression of AGE/RAGE associated signaling proteins and the possible implications that these results could mean for those at risk for developing diabetic cardiovascular complications.

## Figures and Tables

**Table 1:** Physiology measurements of mice used within this study.

	Body Weight (g)	Heart Weight (g)	Blood Glucose (mg/dL)
		Body Weight (g)	
Non-Diabetic (n = 30)	28.87 ± 0.2489	0.0038 ± 4.640 × 10^−5^	195.1 ± 4.932
Diabetic (n = 16)	51.91 ± 0.7320 ****	0.0022 ± 5.594 × 10^−5^ ****	514.2 ± 20.71 ****
Non-Diabetic RKO (n = 24)	31.11 ± 0.4346 **	0.0039 ± 7.490 × 10^−5^	189.9 ± 5.366
Diabetic RKO (n = 14)	54.81 ± 0.7934 ****	0.0024 ± 8.008 × 10^−5^ ****	448.9 ± 20.09 ****
Rap1a KO (n = 27)	27.50 ± 0.3493	0.0040 ± 6.563 × 10^−5^	197.0 ± 6.119

Heart weight, body weight, and non-fasting blood glucose levels were measured before isolation of cardiac fibroblasts. Data presented consists of mean ± SEM. On average, each cardiac fibroblast isolation consisted of 2–3 hearts. Statistical analysis consisted of a one-way ANOVA followed by a Dunnett’s post hoc compared to non-diabetic mice to determine significant differences (** *p* < 0.01, **** *p* < 0.0001).

## Data Availability

All relevant data are included within the manuscript. Supplemental and original blot images are available at: https://figshare.com/s/46773d03689e77f0b35b.

## Supplementary Materials

The following are available online at https://www.mdpi.com/2073-4409/10/3/557/s1, Figure S1: AGE activation of RAGE did not induce changes in protein expression in RKO cardiac fibroblasts, Figure S2: RKO cardiac fibroblasts did not displayed changes in hydrogen peroxide concentrations with increase AGE/RAGE signaling, Figure S3: Diabetic cardiac fibroblasts with functional RAGE exhibited significantly higher concentration of hydrogen peroxide compared to non-diabetic, RKO, and Rap1a KO cells, Figure S4: Under diabetic conditions, Rap1a activity induced changes in p-ERK1/2 but not p-PKC-ζ protein expression levels.
