# Supplementary material for: Rap1a Overlaps the AGE/RAGE Signaling Cascade to Alter Expression of α-SMA, p-NF-κB, and p-PKC-ζ in Cardiac Fibroblasts Isolated from Type 2 Diabetic Mice
Source: Cells. 2021 Mar 4;10(3):557. doi: 10.3390/cells10030557 (PMC8000763; doi:10.3390/cells10030557)
Supplement: Supplementary file 1 [file cells-10-00557-s001.pdf]

cells

MDPI

Supplemantary Information

Rap1a overlaps the AGE/RAGE signaling cascade to alter expression of α-SMA, p-NF-κB, and p-PKC-ζ in cardiac fibroblasts isolated from type 2 diabetic mice?

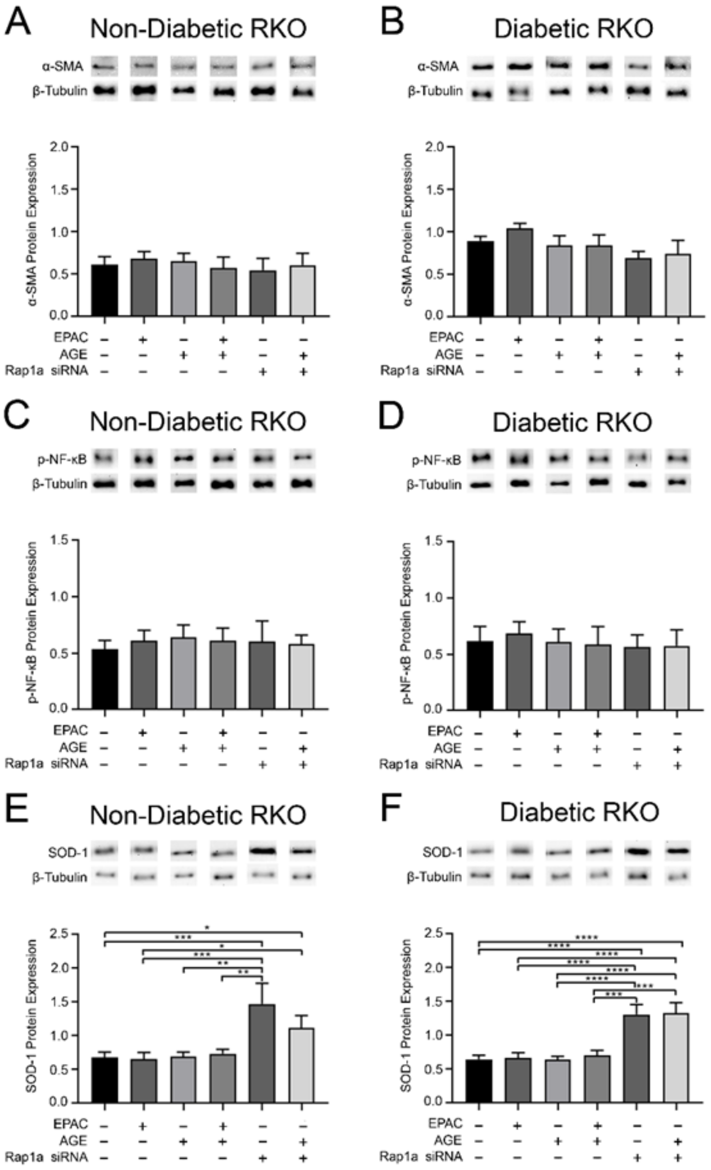

Supplementary Figure 1: AGE activation of RAGE did not induce changes in protein expression in RKO cardiac fibroblasts. Total protein was isolated from non-diabetic RKO and diabetic RKO cardiac fibroblasts and assessed for changes in protein expression. Changes in myofibroblast population was assessed with α-SMA (42 kDa) in (A) non-diabetic RKO and (B) diabetic RKO. Inflammation response was determined by p-NF-κB (65 kDa) in (C) non-diabetic RKO and (D) diabetic RKO fibroblasts. Alterations in oxidative stress was examined by SOD-1 (23 kDa) expression in (E) non-diabetic RKO and (F) diabetic RKO. Fibroblasts were treated with EPAC (100 μM), exogenous AGEs (0.5 mg/mL), and Rap1a siRNA (100 nM) either individually or in combinatorial therapies. Protein

expression was normalized to β-tubulin (55 kDa) protein expression with graphs showing mean ± SEM and n=5-10. Representative western blot images are shown above graphs but are not displayed as a continuous blot due to running order on the blot not aligning with graphical order of samples. Significance was assessed using a one-way ANOVA followed by a Fisher's protected Least Significant Difference post hoc (* p <0.05, ** p <0.01, *** p <0.001, **** p <0.0001).

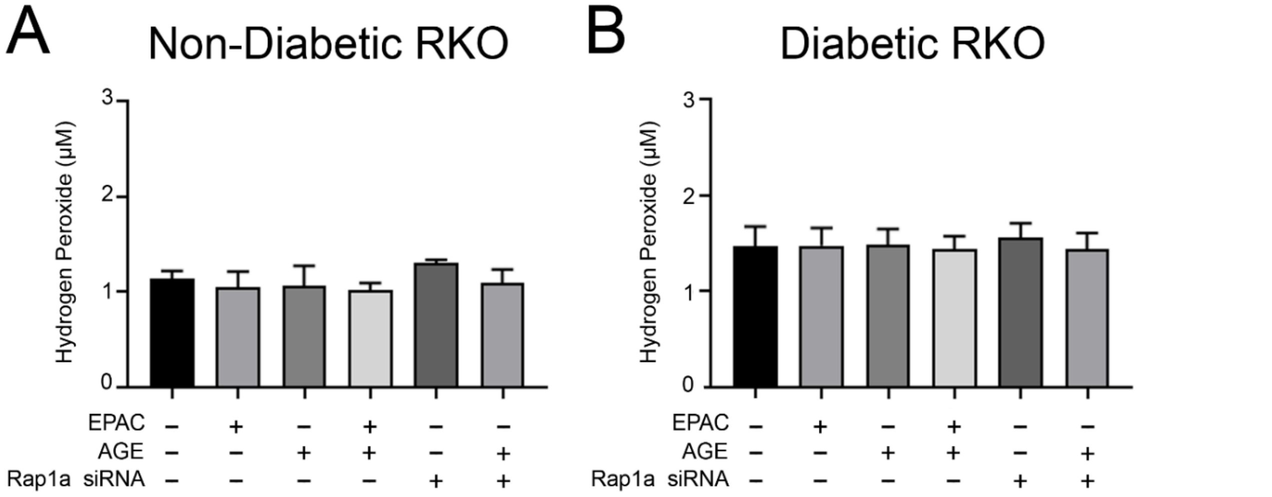

Supplementary Figure 2: RKO cardiac fibroblasts did not displayed changes in hydrogen peroxide concentrations with increase AGE/RAGE signaling. Cardiac fibroblasts isolated from (A) non-diabetic RKO and (B) diabetic RKO mice were treated with different combinations of pharmacological modifiers: EPAC (100 µM), exogenous AGEs (0.5 mg/mL), and Rap1a siRNA (100 nM). The cells were lysed and used to assess the concentration of hydrogen peroxide. Values graphed are mean ± SEM (n= 4-11) and a one-way ANOVA followed by a Fisher's protected Least Significant Difference post hoc was conducted to determine significance.

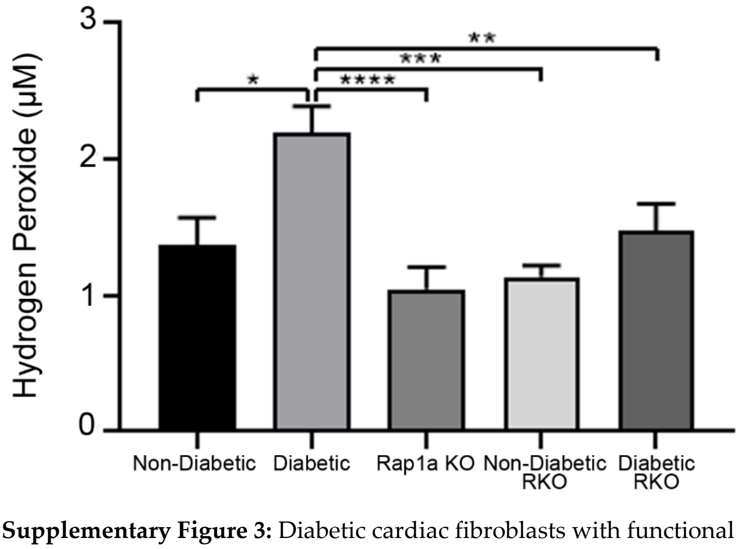

Supplementary Figure 3: Diabetic cardiac fibroblasts with functional RAGE exhibited significantly higher concentration of hydrogen peroxide compared to non-diabetic, RKO, and Rap1a KO cells. Non-diabetic, diabetic, non-diabetic RKO, diabetic RKO, and Rap1a KO mouse hearts were used to isolate cardiac fibroblasts. Untreated cardiac fibroblasts were lysed and utilized to determine concentration of hydrogen peroxide. Values graphed are mean ± SEM (n=4-13) and statistical analysis consisted of a one-way ANOVA followed by a Fisher's protected Least Significant Difference post hoc (* p <0.05, ** p <0.01, *** p <0.001, and **** p <0.0001).

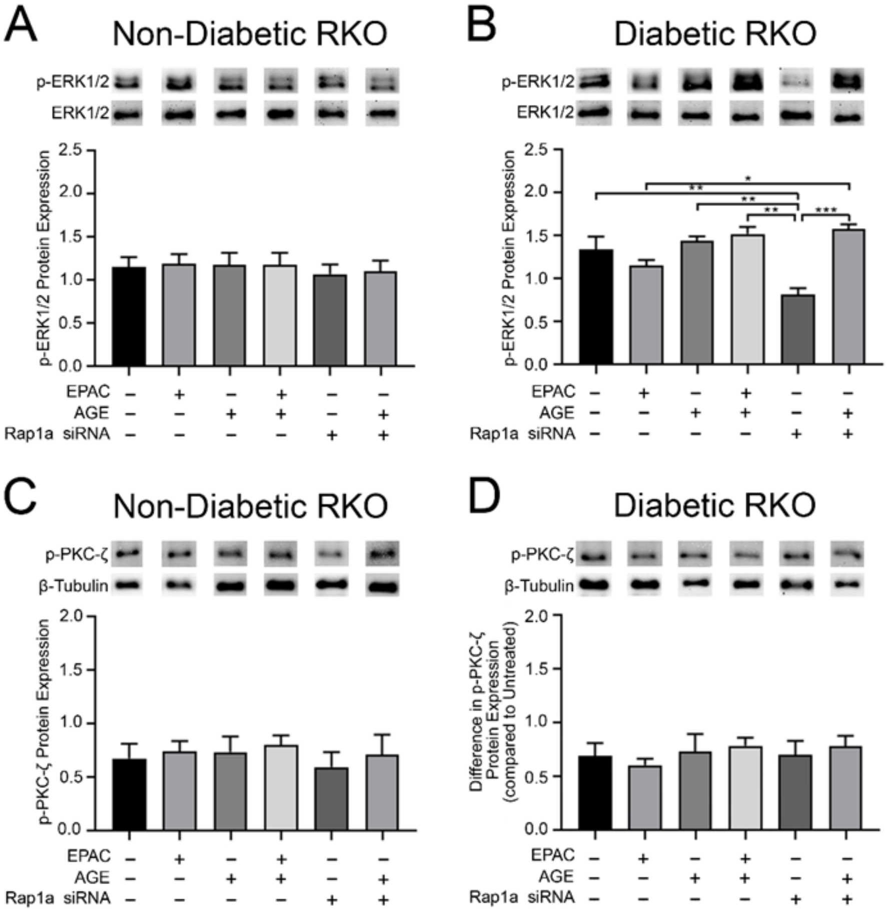

Supplementary Figure 4: Under diabetic conditions, Rap1a activity induced changes in p-ERK1/2 but not p-PKC-ζ protein expression levels. Total protein was isolated from non-diabetic RKO and diabetic RKO cardiac fibroblasts. Fibroblasts were treated with either solo or in combination with EPAC (100 μM), exogenous AGEs (0.05 mg/mL) and/or Rap1a siRNA (100 nM). Changes in protein expression of (A&B) p-ERK1/2 (42 and 44 kDa) and (C&D) p-PKC-ζ (72 kDa) were assessed in the different treatment groups. Protein expression of p-ERK1/2 was normalized to total ERK1/2 (44 and 42 kDa, respectively) protein expression and p-PKC-ζ protein expression was normalized to β-tubulin (55 kDa) protein expression. Mean $\pm$ SEM is depicted in graph with n= 6-8. Representative western blot images are shown above graphs but are not displayed as a continuous blot due to running order on the blot not aligning with graphical order of samples. Significance was assessed using an one-way ANOVA followed by a Fisher's protected Least Significant Difference post hoc (*p<0.05, **p<0.01, ***p<0.001).
